# Expanded CUG Repeat RNA Induces Premature Senescence in Myotonic Dystrophy Model Cells

**DOI:** 10.3389/fgene.2022.865811

**Published:** 2022-03-25

**Authors:** Yuhei Hasuike, Hideki Mochizuki, Masayuki Nakamori

**Affiliations:** Department of Neurology, Osaka University Graduate School of Medicine, Osaka, Japan

**Keywords:** IGFBP3, PAI-1, repeat expansion, cellular senescence, reactive oxygen species, myotonic dystrophy

## Abstract

Myotonic dystrophy type 1 (DM1) is a dominantly inherited disorder due to a toxic gain of function of RNA transcripts containing expanded CUG repeats (CUG^exp^). Patients with DM1 present with multisystemic symptoms, such as muscle wasting, cognitive impairment, cataract, frontal baldness, and endocrine defects, which resemble accelerated aging. Although the involvement of cellular senescence, a critical component of aging, was suggested in studies of DM1 patient-derived cells, the detailed mechanism of cellular senescence caused by CUG^exp^ RNA remains unelucidated. Here, we developed a DM1 cell model that conditionally expressed CUG^exp^ RNA in human primary cells so that we could perform a detailed assessment that eliminated the variability in primary cells from different origins. Our DM1 model cells demonstrated that CUG^exp^ RNA expression induced cellular senescence by a telomere-independent mechanism. Furthermore, the toxic RNA expression caused mitochondrial dysfunction, excessive reactive oxygen species production, and DNA damage and response, resulting in the senescence-associated increase of cell cycle inhibitors p21 and p16 and secreted mediators insulin-like growth factor binding protein 3 (IGFBP3) and plasminogen activator inhibitor-1 (PAI-1). This study provides unequivocal evidence of the induction of premature senescence by CUG^exp^ RNA in our DM1 model cells.

## Introduction

Myotonic dystrophy type 1 (DM1, OMIM #160900) is the most common form of muscular dystrophy in adults (Johnson et al., 2021). The clinical manifestations of DM1 are characterized by multisystemic symptoms, including muscle wasting, myotonia, cardiac conduction defects, cataracts, frontal balding, cognitive impairment, and endocrine defects (Thornton, 2014; Meola and Cardani, 2015). DM1 is caused by the expansion of CTG repeats in the 3′-untranslated region (UTR) of *DMPK* (Brook et al., 1992; Fu et al., 1992; Mahadevan et al., 1992). The RNA expressed from the mutant allele exerts a toxic gain of function due to the presence of an expanded CUG repeat (CUG^exp^), which forms ribonuclear foci in the nucleus and disrupts the regulation of alternative splicing by affecting several RNA-binding factors, including muscleblind-like splicing regulator one and CUGBP Elav-like family member one proteins (Lee and Cooper, 2009). Aberrant splicing events in *CLCN1*, *SCN5A*, and *INSR* have been associated with myotonia, arrhythmias, and insulin resistance, respectively, in DM1 (Savkur et al., 2001; Charlet et al., 2002; Freyermuth et al., 2016). However, although more than hundreds of mis-splicing events have been identified in DM1 tissues (Nakamori et al., 2013; Freyermuth et al., 2016; Otero et al., 2021), the aberrant splicing events responsible for many multisystemic DM1 symptoms remain unclear, suggesting the involvement of additional factors in DM1 pathogenesis.

Some of the multisystemic symptoms in DM1, such as muscle wasting, cataract, cognitive impairment, and frontal baldness, resemble the appearance of accelerated aging (Meinke et al., 2018). Cellular senescence is a crucial driver of the aging process and is defined as a state of irreversible cell cycle arrest induced by stress or specific physiological processes, such as telomere erosion, oncogene overexpression, oxidative stress, mitochondrial dysfunction, and inflammation (Hernandez-Segura et al., 2018; Di Micco et al., 2021). It is characterized by morphological and metabolic changes, chromatin reorganization, and a senescence-associated secretory phenotype (SASP) (McHugh and Gil, 2018). Several studies have shown that DM1 patient-derived cells reduce proliferative capacity and induce cellular senescence (Furling et al., 2001; Bigot et al., 2009; Thornell et al., 2009; Renna et al., 2014). Other studies have suggested the involvement of oxidative stress and mitochondrial dysfunction in DM1 pathogenesis (Sahashi et al., 1992; Usuki and Ishiura, 1998; Siciliano et al., 2001; Toscano et al., 2005; Garcia-Puga et al., 2020). However, the mechanism of cellular senescence in DM1 remains still unclear, because of the limitations in assessing cellular senescence in human primary cells. The characteristics of patient-derived cells vary considerably in different genetic and environmental backgrounds (Chang et al., 2002; Maier and Westendorp, 2009; Dolivo et al., 2016). Furthermore, the proliferative lifespan of human primary cells depends on the donor’s age (Kaji et al., 2009). In this study, we developed a cell model of DM1 conditionally expressing abnormal RNA containing CUG^exp^ to overcome the issue of variability in primary cells of different origins. Then, we investigated whether CUG^exp^ RNA induced cellular senescence and which senescence process was key in DM1 pathogenesis.

## Materials and Methods

### Cell Culture and Transfection

Plasmid pLC16 was used for conditional transcription of expanded CTG repeats, as previously described (Nakamori et al., 2011b). Briefly, pLC16 consists of a cytomegalovirus/chicken β-actin enhancer/promoter, followed by a floxed selection-stop cassette, a downstream complementary DNA (cDNA) sequence for hygromycin resistance, and, finally, the human *DMPK* 3′-UTR, modified with restriction sites for insertion of expanded CTG repeats (Supplementary Figure S1A). The selection-stop cassette contains cDNA encoding a puromycin resistance protein (*puro*) followed by a triple-stop transcription terminator. The expanded CTG repeat was synthesized in the repeat donor plasmid pDWD using cell-free cloning by amplification of dimerized expanded repeats. The expanded CTG repeat was inserted into the *DMPK* 3′-UTR sequence in the pLC16 plasmid construct.

DM1 fibroblasts (GM05281) were purchased from Coriell Institute. DM1 myoblasts were obtained from biopsy specimens as described previously (Nakamori et al., 2017). Human fetal lung fibroblasts (TIG-3 cells, passage 20) were obtained from the Japanese Collection of Research Bioresources Cell Bank. They were cultured in Dulbecco’s modified Eagle medium with low glucose (Gibco) supplemented with 10% fetal bovine serum. Then, the TIG-3 cells were cotransfected with pLC16 containing 800 CTG repeats and phiC31 integrase using Nucleofector Technology with program U-023 (Lonza). Stably transfected cells were selected with puromycin (0.5 μg/ml). The expression of expanded CUG RNA was induced by *Cre* recombinase-mediated excision of the *puro*-transcription-terminator cassette. Cells with recombination were selected using hygromycin B (50 μg/ml). The proliferative capacity of the cells was assessed by calculating CPDL, as previously described (Bigot et al., 2009).

### Fluorescent *in situ* Hybridization (FISH)

FISH was performed as previously described (Nakamori et al., 2011b). The resultant fluorescence images were obtained using a BZ-X710 fluorescence microscope (Keyence).

### SA-β-Gal and BrdU Activity and Proliferative Capacity

SA-β-gal activity was determined using a Senescence Cells Histochemical Staining Kit (Sigma-Aldrich), according to the manufacturer’s protocol. Images were obtained with a BZ-X710 fluorescence microscope. Proliferating cells were detected using a BrdU Immunohistochemistry Kit (Abcam), according to the manufacturer’s protocol. Briefly, the cells were incubated with 10 μM BrdU for 24 h at 37°C. BrdU-positive cells were analyzed using ImageJ software (National Institutes of Health).

### Quantitative RT-PCR

Total RNA was extracted from the DM1 model cells using an RNeasy Mini Kit (Qiagen). Then, the RNA was reverse transcribed to cDNA using a Superscript III First-Strand Synthesis System (Invitrogen), according to the manufacturer’s protocol. qPCR was performed using TaqMan Gene Expression assays on an ABI PRISM 7900HT Sequence Detection System (Applied Biosystems). Gene expression was determined using TaqMan primers and probes for *CDKN1A*, *TP53*, *CDKN2A*, *IGFBP3*, *SERPINE1*, *NQO1*, *HMOX1*, *MMP1*, and *MMP3* (Applied Biosystems). Relative mRNA expression was normalized to 18S rRNA. The level of endogenous *DMPK* plus transgene-derived mRNA was determined as described previously (Nakamori et al., 2011a). Alternative splicing of *MBNL1* exon 5 and *MBNL2* exon 5 was analyzed as described previously (Nakamori et al., 2016).

### RNA-Seq Analysis

Whole transcriptome RNA-seq analysis was performed on CUG^exp−^ and CUG^exp+^ cells using a NovaSeq 6,000 System (Illumina). The raw data were evaluated by NGQC software (Novogene) and trimmed to remove adaptor contaminants and low-quality reads. Clean reads were aligned to the reference genome sequence using TopHat v2.0.12 (Trapnell et al., 2009). The estimated transcript abundance was calculated, and the count values were normalized to the upper quartile of the fragments per kilobase of transcript per million mapped reads using HTSeq v0.6.1 (Anders et al., 2015). GO enrichment analyses were conducted using goseq v2.12.0 (Young et al., 2010) and Metascape (http://www.metascape.org). Sequencing data have been deposited in Gene Expression Omnibus under accession number GSE196265.

### Western Blot

Total cell proteins were prepared from the DM1 model cells, as previously described (Nakamori et al., 2017). Then, the proteins were separated by sodium dodecyl sulfate-polyacrylamide gel electrophoresis and immunoblotted with the following primary antibodies: mouse anti-IGFBP3 (1:500; MAB305, R&D Systems), rabbit anti-PAI-1 (1:2000; NBP1-19773, Novus), rabbit anti-AKT (1:500; GTX121937, GeneTex), rabbit anti-phospho-Akt (Ser473) (1:500; 4,058, Cell Signaling Technology), rabbit anti-p53 (1:100; 2,527, Cell Signaling Technology), rabbit anti-p21 (1:1,000; 2,947, Cell Signaling Technology), rabbit anti-p16 (1:1,000; ab108349, Abcam), and rabbit anti-GAPDH (1:1,000; G9545, Sigma-Aldrich). After incubation, the immunoblots were washed, incubated with horseradish peroxidase-conjugated anti-mouse immunoglobulin (Ig) G or anti-rabbit IgG (GE Healthcare), and detected by ECL Prime Western Blotting Detection Reagent (GE Healthcare) using a ChemiDoc Touch Imaging System (Bio-Rad).

### Enzyme-Linked Immunosorbent Assay for Secreted IGFBP-3

The levels of secreted IGFBP-3 were measured using a Human IGFBP-3 Quantikine ELISA Kit (R&D Systems), according to the manufacturer’s instructions. Recombinant human IGFBP-3 protein (R&D Systems) was used as the standard control.

### Telomere Length Measurement

Genomic DNA was extracted from the DM1 model cells using a Gentra Puregene Cell Kit (Qiagen). Then, telomere length was measured using a Relative Human Telomere Length Quantification qPCR Assay Kit (ScienCell), according to the manufacturer’s protocol. Briefly, the extracted genomic DNA was added to a reaction containing a primer pair (telomere or single copy reference) and Power SYBR Green PCR Master Mix (Applied Biosystems). PCR was performed on an ABI PRISM 7900HT Sequence Detection System (Applied Biosystems).

### Comet Assay

Comet assays were performed, as previously described (Olive and Banath, 2006). Briefly, cell suspensions were mixed with a 1% low-gelling-temperature agarose solution and spotted onto agarose-precoated glass slides. The slides were gently submerged in an alkaline lysis solution for 4 h at 4°C and then transferred to an electrophoresis solution and electrophoresed at 20 V for 25 min. Next, the slides were submerged in rinse buffer for 30 min at room temperature and then incubated with 2.5 μg/ml SYBR Green Ⅰ Nucleic Acid Gel Stain (Invitrogen) for 20 min. The results were analyzed using ImageJ software and scored as the percentage of tail DNA and Olive tail moment.

### Immunofluorescence

The DM1 model cells were plated on Lab-Tek II chamber slides (Thermo Fisher Scientific) and incubated for 24 h. After washing with PBS, the cells were fixed in 4% paraformaldehyde for 15 min and permeabilized with 0.3% Triton for 5 min. Then, cells were blocked with 5% BSA for 30 min and incubated with mouse anti-phospho-Histone H2A.X (Ser139) antibody (1:500; 05-636, Merck Millipore) overnight at 4°C. After washing with PBS, cells were incubated with goat-anti-mouse Alexa 488 secondary antibody (1:500) for 1 h. Cells were washed with PBS and mounted with Vectashield Hardset mounting medium with DAPI (Vector Laboratories). The fluorescence images were obtained using a BZ-X710 fluorescence microscope (Keyence).

### Abasic Site Measurement

The number of AP sites was assessed using a colorimetric DNA Damage Quantification Kit-AP Site Counting (Dojindo), according to the manufacturer’s instructions. The results were measured on a Multiskan FC Microplate Photometer (Thermo Fisher Scientific).

### Flow Cytometry Analysis of ROS Production and Mitochondrial Membrane Potential

ROS production was determined by flow cytometry using MitoSOX Red Mitochondrial Superoxide Indicator (Invitrogen), and the mitochondrial membrane potential was measured by flow cytometry using Rhodamine 123 (Invitrogen). Briefly, the DM1 model cells were seeded at a density of 80% confluency on 12-well plates. Then, H_2_O_2_ (200 µM) was added to induce ROS production, and the cells were incubated at 37°C for 1 h. After detaching the cells from the wells using trypsin, they were incubated with 5 µM MitoSOX or 10 μM Rhodamine 123 for 30 min at 37°C. Finally, the cells were analyzed on a BD FACSCanto II flow cytometer (BD Biosciences). The total cell population was defined according to the forward versus side scatter dot plot, and data for the live cells only were extracted for analysis. The median fluorescence intensity (MFI) of at least 10,000 cells was analyzed using FlowJo software (BD Biosciences). Unstained cells were used as a control.

### ATP Measurement

The levels of cellular ATP were quantified using an Intracellular ATP Assay Kit v2 (TOYO B-Net), according to the manufacturer’s instructions. The emitted luciferin luminescence was quantified using a Glomax 20/20 Luminometer (Promega). Results were corrected for protein concentration using ATP-extracted samples.

### Statistical Analysis

Data were presented as the mean ± standard deviation (SD) from at least three independent biological replicates. Statistical significance was tested using the Student’s *t*-test. *p* values <0.05 were considered statistically significant.

## Results

### Expanded CUG Repeat RNA Induces Cellular Senescence in DM1 Model Cells

We developed a DM1 cell model to investigate the effect of CUG^exp^ RNA on cellular senescence. First, we transfected normal human lung fibroblasts (TIG-3 cells) with plasmid pLC16 containing 800 CTG repeats (day 6). Then, we treated the cells with *Cre* recombinase, and obtained DM1 model cells conditionally expressing CUG^exp^ RNA after 18 days of selection using hygromycin B (day 47). This cell model allowed us to control CUG^exp^ RNA expression in primary cells with the same genetic background so that we could assess the mechanism of cellular senescence by CUG^exp^ RNA expression more accurately than in patient-derived cells.

**FIGURE 1:**
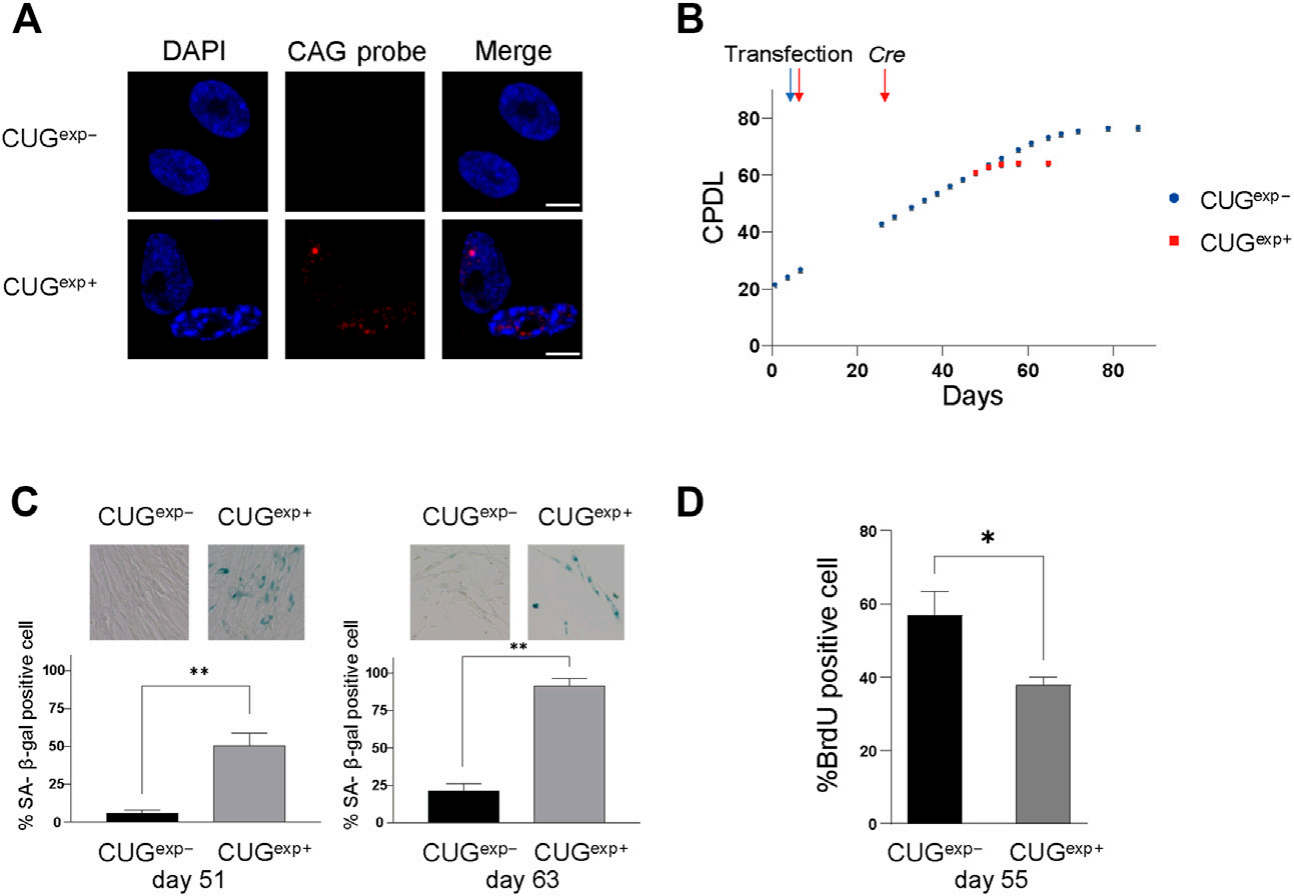
Expanded CUG repeat RNA induces cellular senescence in DM1 model cells. **(A)** Representative images of RNA FISH analysis in cells not expressing CUG^exp^ (CUG^exp−^) and cells expressing CUG^exp^ (CUG^exp+^) at day 53. Nuclear CUG^exp^ foci were present in CUG^exp+^ cells. Scale bar: 10 μm. DAPI: 4′,6-diamidino-2-phenylindole. **(B)** Cumulative population doubling levels (CPDL) of CUG^exp−^ cells (blue) and CUG^exp+^ cells (red) during continuous passages. Cells were transfected with plasmid pLC16 containing 800 CTG repeats at day 6. CUG^exp^ RNA was induced by *Cre* recombinase at day 25 in CUG^exp−^ cells. Data are presented as means ± SD of three independent experiments. **(C)** Senescence-associated β-galactosidase (SA-β-gal) activity in CUG^exp−^ and CUG^exp+^ cells at day 51 (left) and day 63 (right). Representative images of SA-β-gal staining (top). Bar graph shows the percentage of SA-β-gal-positive cells (bottom). Data are presented as means ± SD of three independent experiments. **p < 0.001. **(D)** Immunohistochemical quantification of the percentage of BrdU-positive cells at day 55. Data are presented as means ± SD of three independent experiments. *p < 0.01.

In the cells expressing CUG^exp^ RNA following induction by *Cre* recombinase, the total expression level of transgene and endogenous *DMPK* was increased 3.8-fold compared with that of endogenous *DMPK* and DM1 fibroblasts and to a similar level in DM1 myoblasts (Supplementary Figure S1B). RNA-fluorescence *in situ* hybridization (FISH) experiments showed RNA foci formation, as observed in DM1 patient cells (Figure 1A; Supplementary Figure S1C). Splicing misregulation in *MBNL1* and *MBNL2* was also induced by CUG^exp^ RNA expression (Supplementary Figure S1D). We calculated the cumulative population doubling level (CPDL) by culturing the DM1 model cells to compare the proliferative capacity of cells expressing CUG^exp^ (CUG^exp+^ cells) with those not expressing CUG^exp^ (CUG^exp−^ cells). The induction of abnormal RNA slowed cell proliferation and prematurely terminated cell division (Figure 1B; Supplementary Figure S2A). We also confirmed that the induction of CUG^exp^ RNA reduced the proliferative capacity in two other independently established TIG-3 cell lines conditionally expressing CUG^exp^ RNA (Supplementary Figure S2B). However, the integration sites of the transgene and the expression level of CUG^exp^ RNAs are different in each cell line. Therefore, to avoid the effect of variation in genetic background, we further investigated the mechanism of the premature arrest of cell proliferation in a representative cell line with a sufficient evaluation period after the induction of CUG^exp^ RNA expression in the same genetic background. Furthermore, we confirmed that *Cre* induction and hygromycin B selection did not affect cell proliferation in TIG-3 cells transfected with plasmid pLC16 containing no CTG repeat (Supplementary Figure S2C).

We examined the activity of senescence-associated β-galactosidase (SA-β-gal), a biomarker of senescent cells, to clarify whether the premature arrest was associated with senescence. There were 9.4-fold and 4.3-fold increases in SA-β-gal positivity in CUG^exp+^ cells compared with CUG^exp−^ cells on day 51 and day 63, respectively (*p* = 0.0008 and 0.00006, respectively, Figure 1C). Furthermore, CUG^exp+^ cells showed significantly lower levels of bromodeoxyuridine (BrdU) positivity (indicating cells with proliferative activity) compared with CUG^exp−^ cells (*p* = 0.0083, Figure 1D). Thus, CUG^exp^ expression induced early arrest of cell division and senescence in the DM1 model cells.

### Expanded CUG Repeat RNA Alters Gene Expression Profiles Related to Cellular Senescence

**FIGURE 2:**
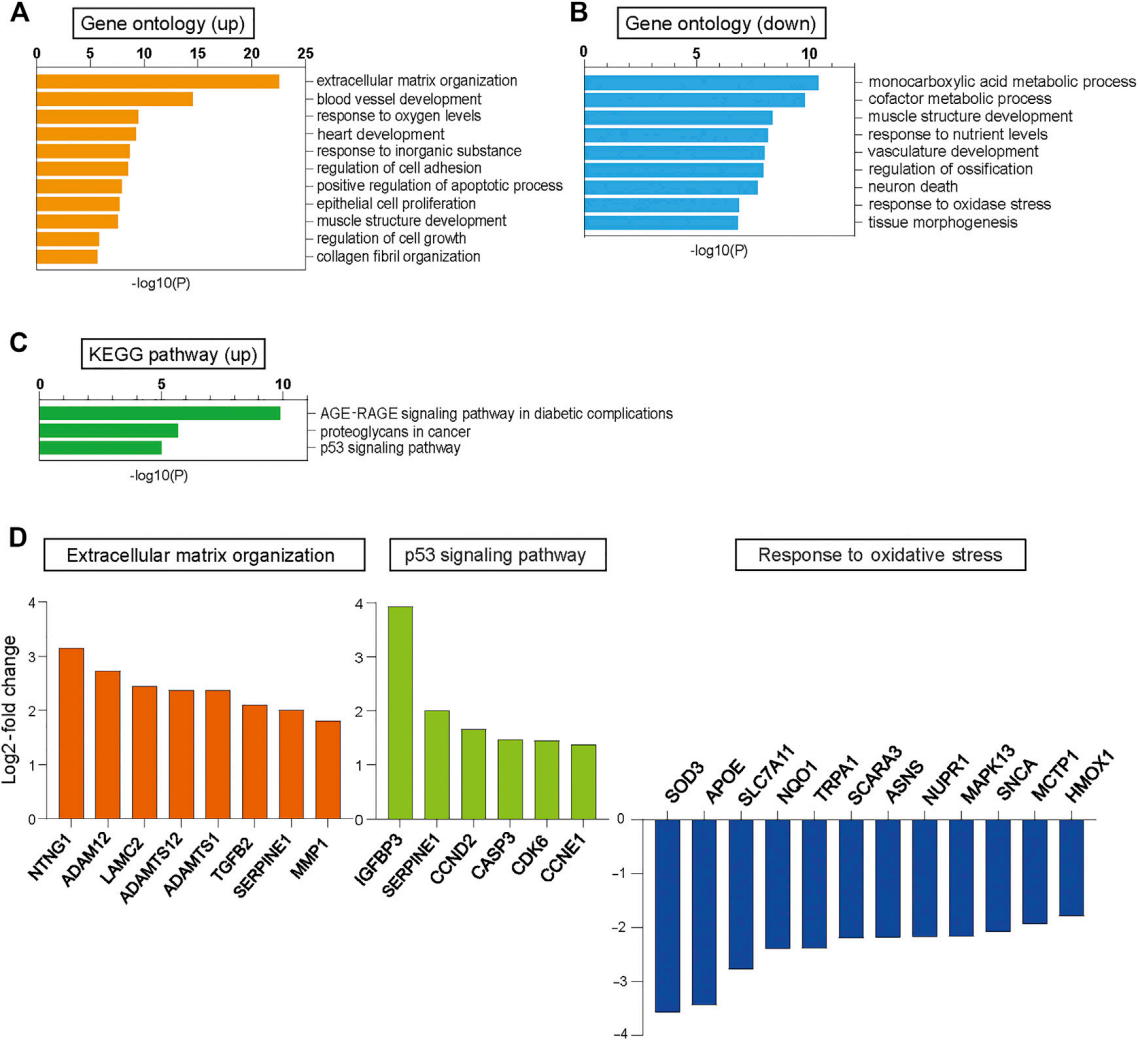
RNA-seq analysis in CUG^exp−^ and CUG^exp+^ cells. **(A)** Bar graphs displaying the GO categories showing significant enrichment among the upregulated genes in cells expressing CUG^exp^ (CUG^exp+^) at day 51. **(B)** Bar graphs displaying the GO categories showing significant enrichment among the downregulated genes in CUG^exp+^ cells at day 51. **(C)** Bar graphs displaying the Kyoto Encyclopedia of Genes and Genomes (KEGG) pathways showing significant enrichment for upregulated genes in CUG^exp+^ cells. **(D)** Expression levels of representative upregulated or downregulated genes in CUG^exp+^ cells involved in ECM organization (orange), p53 signaling pathway (green), and response to oxidative stress (blue).

**FIGURE 3:**
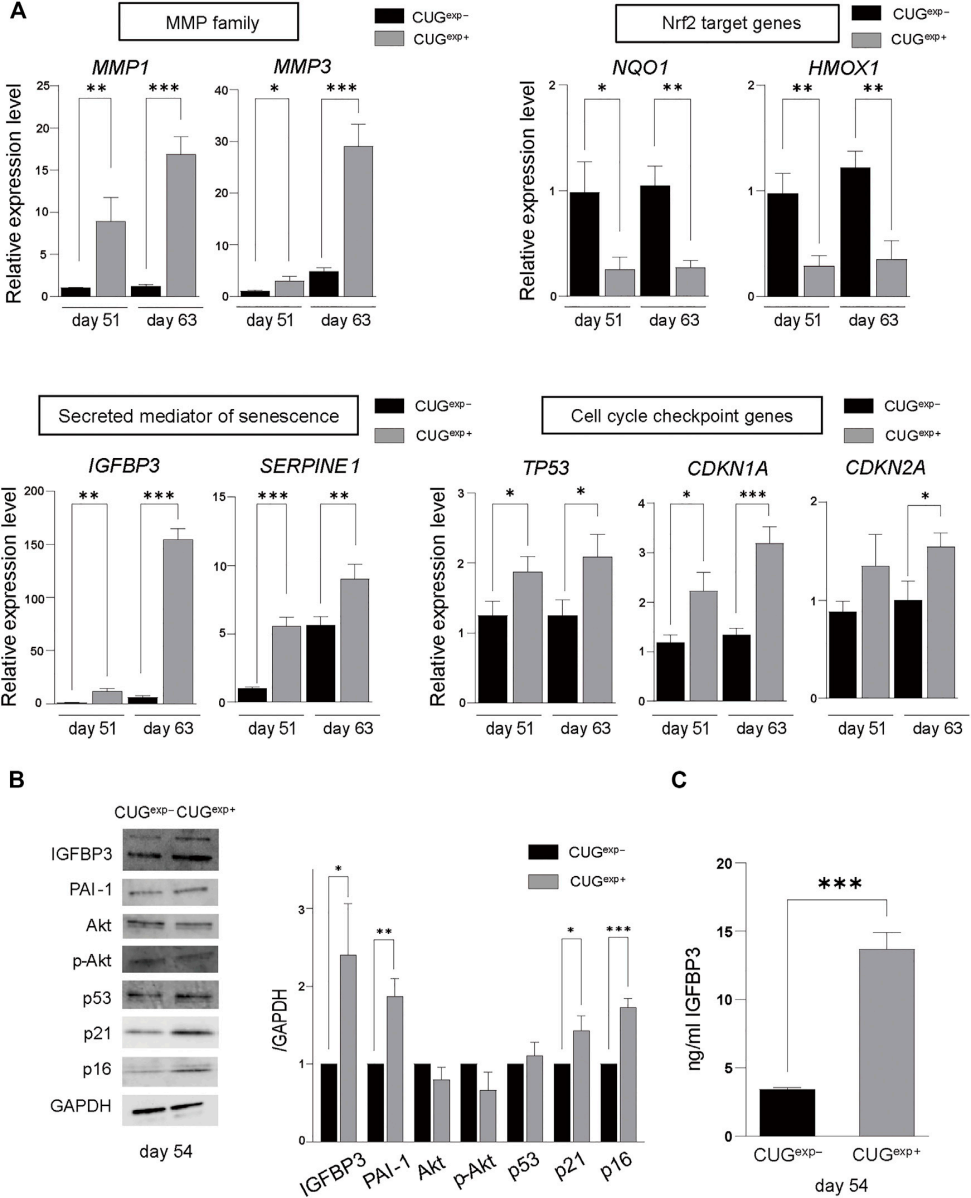
Expanded CUG repeat RNA alters gene and protein expression levels related to cellular senescence. **(A)** Gene expression levels of MMP family genes (*MMP1* and *MMP3*) (top left), Nrf2 target genes (*NQ O 1* and *HMOX1*) (top right), secreted mediators of senescence genes (*IGFBP3* and *SERPINE1*) (bottom left), and cell cycle checkpoint genes (*TP53*, *CDKN1A*, and *CDKN2A*) (bottom right) determined by qRT-PCR in cells not expressing CUG^exp^ (CUG^exp−^) and cells expressing CUG^exp^ (CUG^exp+^) cells at days 51 and 63. Values are presented as means ± SD of three independent experiments. *p < 0.05, **p < 0.01, and ***p < 0.001. **(B)** Representative images of western blots of IGFBP3, PAI-1, Akt, phospho-Akt (Ser437), p53, p21, and p16 proteins in CUG^exp−^ and CUG^exp+^ cells at day 54 (left). GAPDH was used as the loading control. Bar graph shows quantification of the immunoblot (right). Values are presented as means ± SD of three independent experiments. *p < 0.05, **p < 0.01, and ***p < 0.001. **(C)** IGFBP3 levels in the culture medium of CUG^exp−^ and CUG^exp+^ cells at day 54. Values are presented as means ± SD of three independent experiments. ***p < 0.001.

We performed RNA sequencing (RNA-seq) analysis in CUG^exp+^ and CUG^exp−^ cells at day 51 to identify the pathway that induced cellular senescence when CUG^exp^ RNA was expressed. Up-regulated and down-regulated genes in CUG^exp+^ cells had some overlap with those in DM1 myoblasts or myotubes reported in a previous study, despite the cell type difference (Todorow et al., 2021) (Supplementary Table S1). Gene ontology (GO) enrichment analysis was performed to identify relevant biological pathways. Genes for extracellular matrix (ECM) organization, positive regulation of the apoptotic process, and regulation of cell growth were highly enriched among the upregulated genes in CUG^exp+^ cells (Figure 2A). The genes involved in ECM organization included *MMP1* and *ADAMT* genes (Figure 2D). These genes encode ECM-degrading enzymes, which are important molecules comprising the SASP (Mavrogonatou et al., 2019). Among the ECM-degrading enzymes, the matrix metalloproteinase (MMP) family is predominantly linked with cellular senescence (Mavrogonatou et al., 2019). For example, *MMP1* and *MMP3* expression was reported as upregulated in senescent fibroblasts (Coppe et al., 2010). We evaluated *MMP1* and *MMP3* expression in the DM1 model cells at days 51 and 63 by quantitative reverse transcription-polymerase chain reaction (qRT-PCR) and found that the expression of these genes was significantly increased in CUG^exp+^ cells (*p* = 0.0087 and 0.023 at day 51, and *p* = 0.0002 and 0.0007 at day 63, respectively, Figure 3A), suggesting that CUG^exp^ RNA alters the expression of several ECM-degrading enzymes related to cellular senescence. Genes for muscle structure development and response to oxidative stress were significantly enriched among the downregulated genes (Figure 2B). The genes involved in the response to oxidative stress and antioxidant expression, such as *NQO1* and *HMOX1*, were significantly decreased (*p* = 0.016 and 0.005 at day 51, and *p* = 0.0026 and 0.0031 at day 63, respectively, Figures 2D, 3A).

Furthermore, Kyoto Encyclopedia of Genes and Genomes (KEGG) pathway analysis showed that the p53 signaling pathway was highly enriched among the upregulated genes in CUG^exp+^ cells (Figure 2C). This pathway involves *SERPINE1*, encoding plasminogen activator inhibitor-1 (PAI-1), and *IGFBP3*, a downstream target of PAI-1-induced senescence, which were both markedly upregulated (*p* = 0.0003 and 0.0014 at day 51, and *p* = 0.0095 and 0.00002 at day 63, respectively, Figures 2D, 3A). These genes were reported not only as markers of senescence but also as inducers of senescence in human and mouse fibroblasts (Debacq-Chainiaux et al., 2008; Vaughan et al., 2017). The protein levels of PAI-1 and insulin-like growth factor binding protein 3 (IGFBP3) were also significantly increased in CUG^exp+^ cells at day 54 (*p* = 0.0026 and 0.021, respectively, Figure 3B). We then assessed the activity of Akt, a critical downstream target downregulated by PAI-1 and IGFBP3 (Balsara et al., 2006; Elzi et al., 2012). Akt phosphorylation at Ser473 was mildly decreased in CUG^exp+^ cells at day 54, although this was not statistically significant (*p* = 0.065, Figure 3B). IGFBP3 has also been reported as a secreted mediator of cellular senescence with paracrine and autocrine activity (Vassilieva et al., 2020). The IGFBP3 levels in the culture medium of the DM1 model cells showed a 4-fold increase in CUG^exp+^ cell cultures at day 54 (*p* = 0.00013, Figure 3C). These results indicated that the PAI-1-IGFBP3 pathway was activated by CUG^exp^ RNA expression in the DM1 model cells.

### Expanded CUG Repeat RNA Activates Cell Cycle Checkpoint Inhibitors

PAI-1 and IGFBP3 were reported as induced by p53 activation (Grimberg et al., 2005; Vaughan et al., 2017). p53 is activated in response to DNA damage and various stressors that characteristically promote cellular senescence to regulate the cell cycle (Di Micco et al., 2021). Cell cycle checkpoint genes, such as *TP53* encoding p53, *CDKN1A* encoding p21, and *CDKN2A* encoding p16, induce cellular senescence despite the presence of metabolic activity (Hernandez-Segura et al., 2018; McHugh and Gil, 2018). To investigate the influence of CUG^exp^ RNA on cell cycle arrest, we measured the expression levels of cell cycle-related genes by qRT-PCR in CUG^exp−^ and CUG^exp+^ cells at days 51 and 63. *TP53* and *CDKN1A* were significantly increased in CUG^exp+^ cells (*p* = 0.021 and 0.011 at day 51, and *p* = 0.021 and 0.0009 at day 63, respectively, Figure 3A). *CDKN2A* was mildly increased in CUG^exp+^ cells at day 51 (*p* = 0.076, Figure 3A) and significantly increased at day 63 (*p* = 0.017, Figure 3A). The protein levels of p21 and p16 were significantly increased in CUG^exp+^ cells at day 54 (*p* = 0.018 and 0.00043, respectively, Figure 3B). p53 was also mildly increased in CUG^exp+^ cells, although the increase did not reach statistical significance (*p* = 0.33, Figure 3B). p21 and p16 are sufficient to establish cell cycle arrest in an independent and interdependent manner (McHugh and Gil, 2018; Di Micco et al., 2021). Our results indicated that CUG^exp^ RNA expression increased the expression of cell cycle checkpoint inhibitors, resulting in cell cycle arrest in the DM1 model cells.

### Expanded CUG Repeat RNA Causes DNA Damage in a Telomere-Independent Manner

**FIGURE 4:**
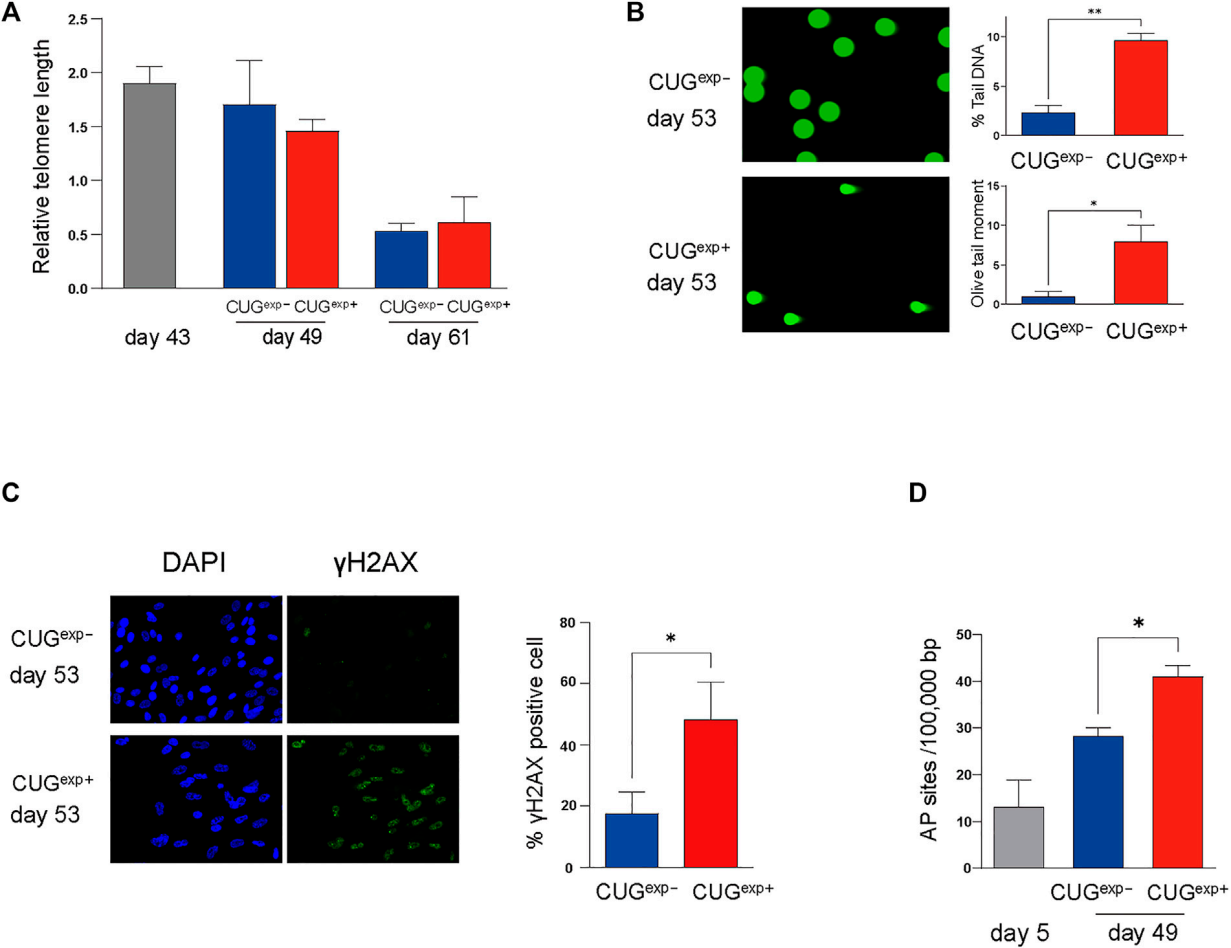
Expanded CUG repeat RNA causes DNA damage in a telomere-independent manner. **(A)** Measurement of relative telomere length by qPCR. Telomere length was measured at three different stages (at days 43, 49, and 61) in cells not expressing CUG^exp^ (CUG^exp−^) (blue) and cells expressing CUG^exp^ (CUG^exp+^) (red). Data are presented as means ± SD of three independent experiments. **(B)** Representative images of DNA damage in comet assays at day 53 (left). DNA damage was evaluated according to the percentage tail DNA and Olive tail moment (right). Data are presented as means ± SD of three independent experiments. *p < 0.01, **p < 0.001. **(C)** Representative images of DNA damage in γ-H2AX assays at day 53 (left). Bar graph showing percentage of γ-H2AX positive cells (right). Data are presented as means ± SD of three independent experiments. *p < 0.05. **(D)** Quantification of DNA damage response by AP site measurement. The AP site lesions per 100,000 base pairs were measured in the cells before transfection (day 5) and at day 49 in cells not expressing CUG^exp^ (CUG^exp−^) and cells expressing CUG^exp^ (CUG^exp+^). Data are presented as means ± SD of three independent experiments. *p < 0.01.

Next, we investigated the regulatory mechanism responsible for the increased levels of cell cycle inhibitors p21 and p16 and secreted mediators IGFBP3 and PAI-1 in the DM1 model cells. Telomere shortening is an important molecular mechanism of cell cycle arrest (Di Micco et al., 2021). Shortened telomeres are recognized as DNA breaks, and they promote cell cycle arrest, known as replicative senescence (Hernandez-Segura et al., 2018; Di Micco et al., 2021). We used qPCR to measure the telomere length in the DM1 model cells at days 43, 49, and 61 to confirm whether the cellular senescence induced by CUG^exp^ occurred in a telomere-dependent manner. Although the telomere length in CUG^exp−^ and CUG^exp+^ cells was shortened in later passages, there was no significant difference in the telomere length between both cell types (Figure 4A). This suggested that telomere shortening was not associated with accelerated cellular senescence caused by the toxic CUG^exp^ RNA.

Telomere-independent senescence (premature senescence) occurs when DNA damage response (DDR) is triggered in response to DNA damage, even in normal proliferating cells (Di Micco et al., 2021). We performed a comet assay on the DM1 model cells to evaluate the DNA damage induced by CUG^exp^. The parameters reflecting the extent of DNA damage, such as the percentage tail DNA and Olive tail moment (OTM), were significantly increased in CUG^exp+^ cells at day 53 (*p* = 0.00022 and 0.0056, respectively, Figure 4B). Furthermore, γ-H2AX foci, indicative of DNA damage, were significantly increased in CUG^exp+^ cells (*p* = 0.019, Figure 4C). Next, we evaluated apurinic/apyrimidinic (AP) sites (markers of base excision repair) to confirm DDR activation in response to DNA damage in the DM1 model cells. The AP site lesions were increased in later passages and were more significantly increased in CUG^exp+^ cells at day 49 (*p* = 0.0015, Figure 4D), suggesting that abnormal CUG^exp^ RNA increased DNA damage and DDR in the DM1 model cells.

### Expanded CUG Repeat RNA Impairs Mitochondrial Function and Increases Reactive Oxygen Species

**FIGURE 5:**
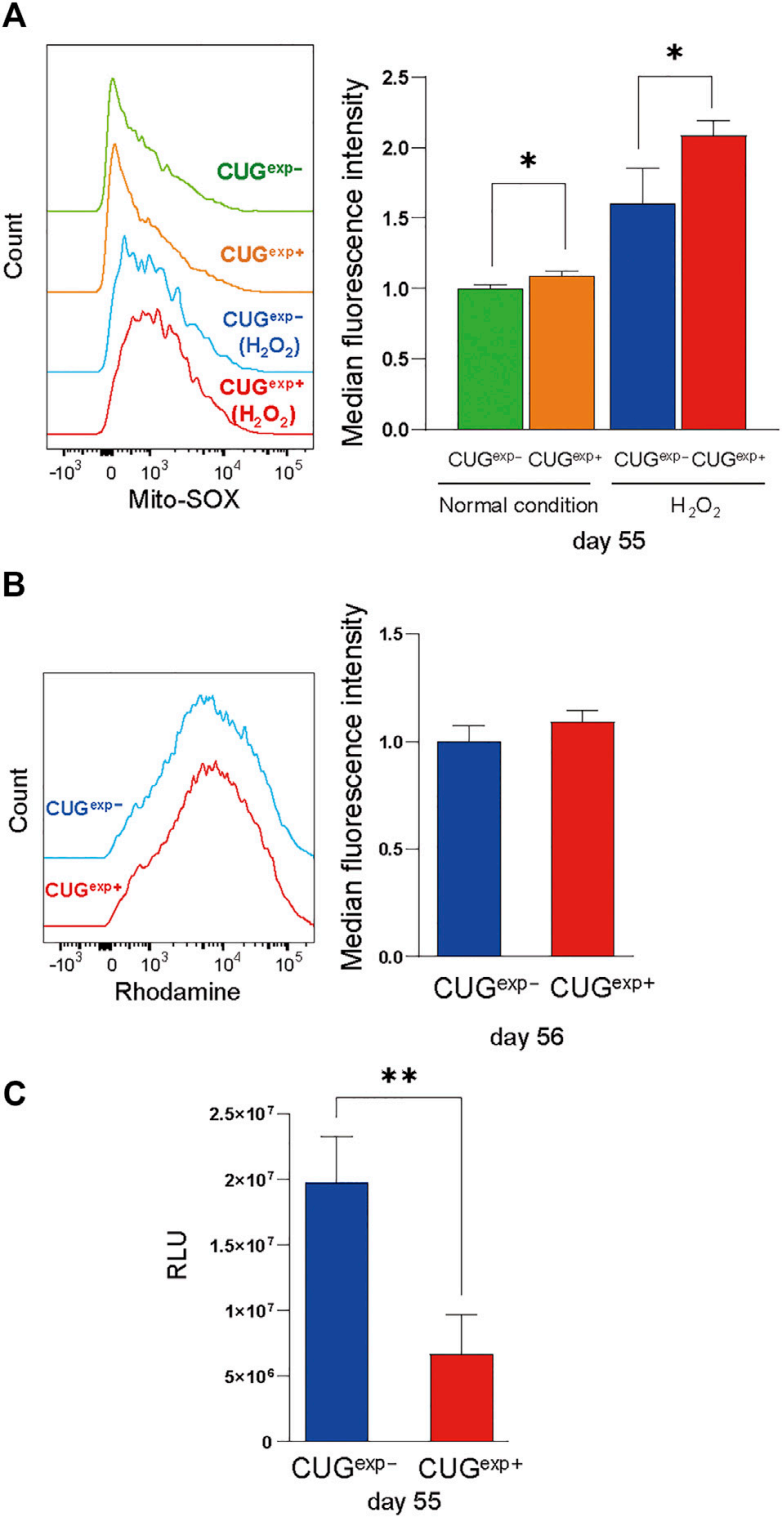
Expanded CUG repeat RNA impairs mitochondrial function and increases ROS. **(A)** Left: Flow cytometry histograms of mitochondrial ROS in cells not expressing CUG^exp^ (CUG^exp−^) (green) and cells expressing CUG^exp^ (CUG^exp+^) (orange) at day 55 under normal conditions and following H_2_O_2_ exposure (CUG^exp−^, blue, and CUG^exp+^, red). Right: Bar graph showing MFI values for mitochondrial ROS. Data are presented as means ± SD of three independent experiments. *p < 0.05. **(B)** Left: Flow cytometry histograms of Rhodamine 123 in CUG^exp−^ (blue) and CUG^exp+^ cells at day 56 (red). Right: Bar graph showing MFI values for Rhodamine 123. Data are presented as means ± SD of three independent experiments. **(C)** Quantification of intracellular ATP production by firefly luciferase luminescence at day 55. Data are presented as means ± SD of three independent experiments. **p < 0.01. RLU: relative light units.

We then investigated the cause of DNA damage in CUG^exp+^ cells. ROS are important in the induction of cellular senescence because they damage DNA *via* a variety of mechanisms, including oxidized DNA bases, AP sites, and double-strand breaks (Davalli et al., 2016). Considering the possibility that excessive ROS production may cause the DNA damage by toxic repeat RNA, we measured mitochondrial ROS by flow cytometry. H_2_O_2_, which induces intracellular ROS generation, was added to evaluate the response to oxidative stress (Weng et al., 2018). In our study, mitochondrial ROS were significantly increased in CUG^exp+^ cells at day 55 under normal conditions, and the difference was more pronounced following H_2_O_2_ exposure (*p* = 0.018 and 0.036, respectively, Figure 5A).

Mitochondria are the major sites of ROS production (Turrens, 2003) and mitochondrial dysfunction contributes to ROS overproduction (Korolchuk et al., 2017). Therefore, we evaluated mitochondrial dysfunction as a cause of ROS production. Rhodamine 123, which accumulates in activated mitochondria that have a high membrane potential, was unaffected by CUG^exp^ RNA expression (Figure 5B). However, although the mitochondrial membrane potential remained unchanged, intracellular ATP levels were significantly decreased in CUG^exp+^ cells at day 55 (*p* = 0.0082, Figure 5C). Decreased cellular ATP production results from mitochondrial bioenergetic dysfunction, and insufficient mitochondrial energy production leads to excessive ROS production (Korolchuk et al., 2017). Thus, our results indicated that CUG^exp^ RNA caused mitochondrial dysfunction, contributing to premature senescence *via* ROS production.

## Discussion

**FIGURE 6:**
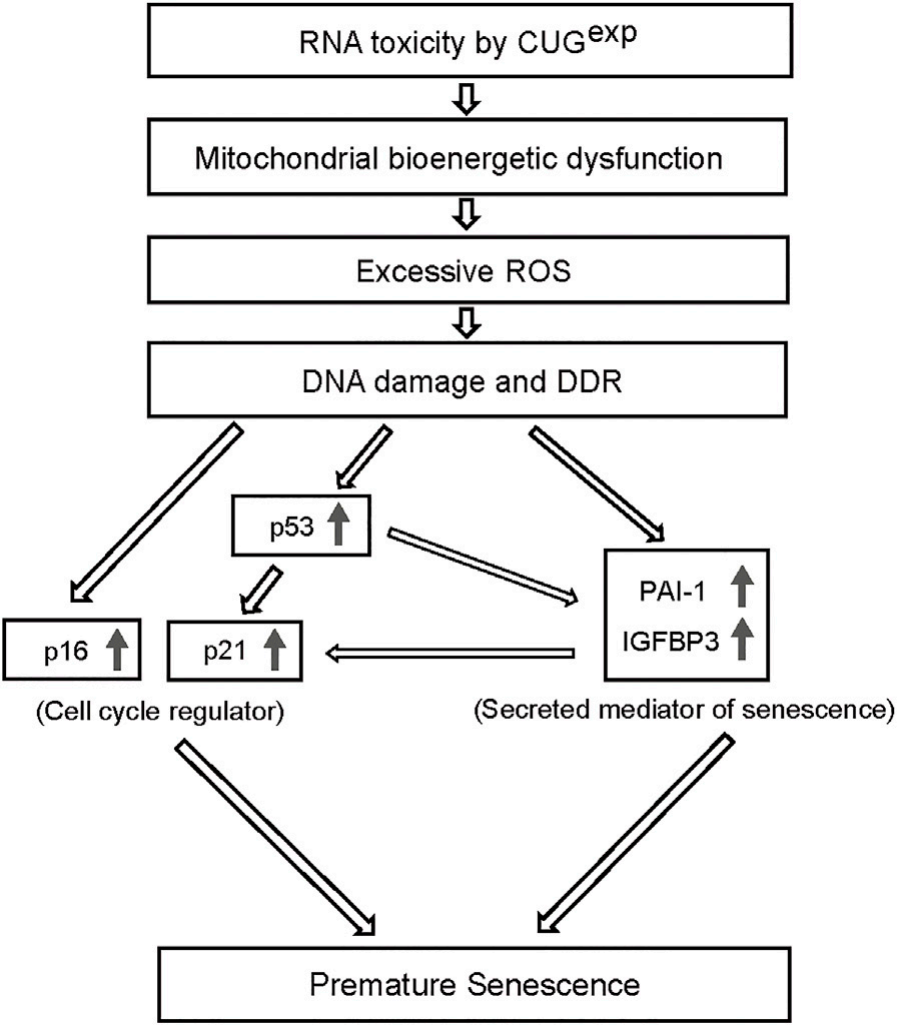
Proposed mechanism of premature senescence by CUG^exp^ RNA expression. RNA toxicity by CUG^exp^ causes mitochondrial dysfunction, which contributes to ROS overproduction, triggering DNA damage and DDR. Accumulated DNA damage activates cell cycle regulators, such as p53, p21, and p16. ROS-induced DNA damage upregulates the secreted mediators PAI-1 and IGFBP3 in a p53-dependent and -independent manner, which drive premature senescence. These multiple steps in the process of senescence interact with one another, inducing premature senescence.

The presentation of multisystemic symptoms that resemble features of aging in patients with DM1, such as muscle wasting, cataract, cognitive impairment, and frontal baldness, suggests DM1 as a progeroid disorder (Meinke et al., 2018). Although several studies of DM1 patient-derived cells indicate the involvement of cellular senescence (Furling et al., 2001; Bigot et al., 2009; Thornell et al., 2009; Renna et al., 2014), the senescence process remains unelucidated. In this study, we established a primary cell model of DM1 with the conditional expression of CUG^exp^ RNA. We demonstrated that CUG^exp^ expression induced premature senescence in the DM1 model cells. Additionally, our results suggest that premature senescence in the DM1 model cells is associated with mitochondrial dysfunction and ROS production, leading to the upregulation of secreted mediators IGFBP3 and PAI-1 and cell cycle regulators p16, p21, and possibly p53 (Figure 6).

Cellular senescence is defined as the permanent cessation of cell proliferation. It is induced by two distinct mechanisms: replicative senescence (triggered by telomere loss) and premature senescence (caused by a telomere-independent mechanism) (Kuilman et al., 2010). Our results suggest that the expression of CUG^exp^ RNA induces cellular senescence regardless of telomere length, which correlates with the findings of another study reporting reduced proliferative capacity in muscle satellite cells from patients with DM1, even though the telomeres did not reach a critical size (Bigot et al., 2009).

Telomere-independent premature senescence is triggered by various cellular stressors, including oxidative stress, oncogene activation, and DNA damage agents such as ionizing radiation or chemotherapeutic substances (Kuilman et al., 2010; Hernandez-Segura et al., 2018). Exposure to these stressors induces persistent DNA damage and DDR, resulting in premature senescence. Our study focused on oxidative stress, a typical internal factor that causes premature senescence, because the use of our DM1 cell model allowed us to exclude the influence of external factors. Oxidative stress is characterized by ROS overproduction (Davalli et al., 2016). ROS directly damage DNA, causing the induction and maintenance of cellular senescence (Passos et al., 2010; Davalli et al., 2016). The majority of ROS are produced in mitochondria, and mitochondrial ROS production increases with mitochondrial metabolic dysfunction (Turrens, 2003; Korolchuk et al., 2017). Mitochondrial ROS were slightly but significantly increased in CUG^exp+^ cells solely by induction of abnormal RNA expression even in physiological condition. Previous studies demonstrated that even slight changes in mitochondrial ROS production are associated with DNA damage and mitochondrial dysfunction in fibroblasts (Kobashigawa et al., 2015; Lofaro et al., 2020). Our findings of increased mitochondrial ROS, decreased intracellular ATP, and DDR activation in CUG^exp+^ cells indicate that CUG^exp^ RNA promotes ROS-mediated premature senescence by mitochondrial dysfunction.

ROS homeostasis is maintained by antioxidants. Nuclear factor-erythroid 2 related factor 2 (Nrf2) transcriptionally regulates several antioxidant genes, such as *HMOX1* and *NQO1*, to decrease ROS levels (Kasai et al., 2020). On the other hand, excess ROS activates p53, which suppresses the Nrf2-regulated antioxidant genes (Drane et al., 2001; Faraonio et al., 2006). Nrf2-dependent antioxidant factors were decreased in our CUG^exp+^ DM1 model cells, suggesting that Nrf2, which is normally activated to eliminate excessive ROS, is suppressed by p53, exacerbating the vulnerability of our DM1 model cells to oxidative stress. Moreover, oxidative stress has been clinically associated with cataracts (Pendergrass et al., 2005), frontal hair loss (Shin et al., 2013), and metabolic dysfunction (de Almeida et al., 2017), which are commonly observed in patients with DM1. Furthermore, antioxidant capacity has been reported as impaired in DM1 patients’ serum (Toscano et al., 2005; Kumar et al., 2014). These findings suggest that ROS-induced premature senescence leads to the features of aging observed in DM1.

The mechanism of cell cycle arrest by CUG^exp^ RNA is largely unknown. The cell cycle is chiefly regulated by the p53-p21 and p16-pRb pathways. Although these pathways have complex interactions, p16 is mainly involved in maintaining cellular senescence by mitogenic stress and sustained DDR (Mijit et al., 2020; Di Micco et al., 2021). Muscle satellite cells derived from patients with congenital DM1 have been reported to induce p16-dependent premature senescence (Bigot et al., 2009). In our study, p16 and p21 expression was significantly increased in CUG^exp+^ cells at both the transcript and protein levels. Moreover, p53 expression was significantly increased in CUG^exp+^ cells at the transcript level and mildly increased at the protein level. The p53-p21 pathway, which is necessary for senescence induction, is upregulated following DDR activation (Mijit et al., 2020; Di Micco et al., 2021). Thus, our data indicate that abnormal CUG^exp^ RNA can induce senescence by activating the p53-p21 pathway and cause irreversible senescence by p16 upregulation.

PAI-1 and IGFBP3 are major factors affecting cellular senescence, mainly as downstream targets of p53 (Grimberg et al., 2005; Vaughan et al., 2017; Vassilieva et al., 2020). In our study, the transcript and protein levels of PAI-1 and IGFBP3 were significantly increased in CUG^exp+^ cells. PAI-1 and IGFBP3 are regulated in both p53-dependent and -independent manner in response to ROS-induced DNA damage (Grimberg et al., 2005; Eren et al., 2014). Hence, CUG^exp^ RNA may increase the expression of PAI-1 and IGFBP3 not only through p53 but also through another signaling pathway activated by ROS-induced DNA damage. Further, PAI-1 and IGFBP3 induce cellular senescence with activation of cell cycle inhibitors. For example, IGFBP3 overexpression upregulates p21 in some cancer cells (Wu et al., 2013), and inhibition of PAI-1 activity reduces p16 expression (Eren et al., 2014). Therefore, the excessive ROS, DNA damage, and DDR observed in CUG^exp+^ cells may increase PAI-1 and IGFBP3, leading to premature senescence *via* activation of cell cycle inhibitors. Additionally, Akt is involved in PAI-1- and IGFBP3-induced cell growth suppression and cellular senescence (Balsara et al., 2006; Elzi et al., 2012). We observed a slight reduction in Akt activity in CUG^exp+^ cells, which is consistent with a previous study using fibroblasts from DM1 patients (Garcia-Puga et al., 2020). Our results suggest the possibility that abnormal CUG^exp^ RNA can inactivate Akt, causing PAI-1- and IGFBP3-mediated senescence. Furthermore, recent studies have shown the suppression of Akt signaling in DM1 skeletal muscle, so reduced Akt activity may be associated with skeletal muscle atrophy (Crawford Parks et al., 2017; Sabater-Arcis et al., 2020; Ozimski et al., 2021). Skeletal muscle atrophy is generally associated with mitochondrial ROS (Powers, 2014), and PAI-1 inhibits the regeneration of damaged skeletal muscle (Rahman and Krause, 2020). Thus, the mechanism of cell proliferation inhibition via PAI-1 and IGFBP3 by ROS-induced DNA damage may also contribute to muscle atrophy in DM1. However, it should be noted that the mechanism of premature senescence observed in our DM1 model fibroblast cells could be different in DM1 myoblast cells. Even in myoblasts, a different mechanism of premature senescence was reported in DM1 and DM2 (Renna et al., 2014).

Previous studies have suggested that cellular senescence in DM1 is associated with mitochondrial dysfunction, oxidative stress, DNA damage, and activation of cell cycle inhibitors (Hasuike et al., 2022). Our study demonstrated the direct effects of abnormal CUG^exp^ RNAs on these factors by using the DM1 model cells conditionally expressing CUG^exp^. However, we were unable to evaluate either the characteristics of CUG^exp+^ cells at a pre-senescence stage or the therapeutic effect by targeting CUG^exp^ RNA or ROS, since CUG^exp+^ cells exhibited delayed cell proliferation soon after the cell model establishment (day 47). Hence, it is not clearly determined whether the senescence inducers such as mitochondrial dysfunction and ROS production observed in CUG^exp+^ cells are the cause or result of premature senescence. Generally, senescence occurs in a bidirectional manner and via various positive feedback mechanisms. For example, cell cycle regulators, such as p53, p21, and p16, produce ROS as a downstream signal transduction factor without oxidative DNA damage, while ROS itself induces p53 (Davalli et al., 2016). Furthermore, the positive feedback loops of mitochondrial damage, ROS production, and DDR activation via the p53-p21 pathway are necessary and sufficient to maintain cell cycle arrest (Passos et al., 2010). Our data suggest that the activation of these positive feedback loops maintains senescence in CUG^exp+^ cells. Several studies have shown impaired mitochondrial function in DM1 (Gramegna et al., 2018; Garcia-Puga et al., 2020). Thus, excessive ROS production by mitochondrial metabolic dysfunction may trigger the series of senescence pathways induced by CUG^exp^ RNA.

In conclusion, our findings indicate that abnormal expanded CUG repeat RNA leads to mitochondrial dysfunction, ROS production, and DDR activation, and induces premature senescence. Various drug targets have been investigated to eliminate the influence of senescent cells and SASP (Di Micco et al., 2021). Interventions targeting the senescence-inducing factors resulting from CUG^exp^ RNA may be potential therapeutics for symptoms that resemble accelerated aging in DM1.

## Data Availability

The datasets presented in this study can be found in online repositories. The names of the repository/repositories and accession number(s) can be found below: https://www.ncbi.nlm.nih.gov/, accession ID: GSE196265.
