# Supplementary material for: Expanded CUG Repeat RNA Induces Premature Senescence in Myotonic Dystrophy Model Cells
Source: Front Genet. 2022 Mar 25;13:865811. doi: 10.3389/fgene.2022.865811 (PMC8990169; doi:10.3389/fgene.2022.865811)
Supplement: Supplementary file 1 [file DataSheet2.PDF]

Figure S2

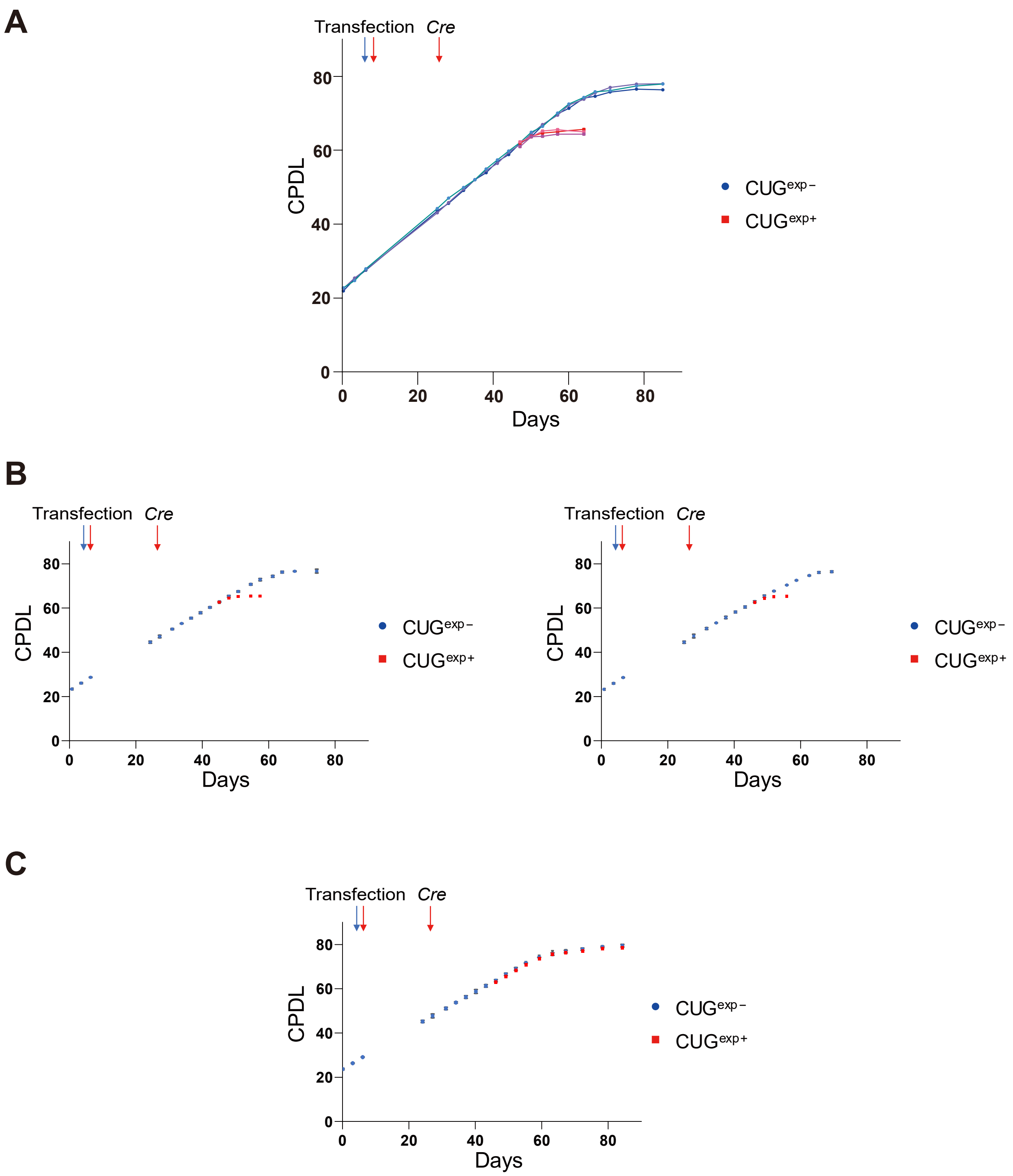

Supplemental Figure S2

(A) Cumulative population doubling levels (CPDL) of CUG^{exp-} cells (blue) and CUG^{exp+} cells (red) during continuous passages in each experiment in Figure 1B. **(B)** CPDL of CUG^{exp-} cells (blue) and CUG^{exp+} cells (red) in two other independently established DM1 model cell lines. Data are presented as means ± SD of three independent experiments. **(C)** CPDL of TIG-3 cells transfected with plasmid pLC16 containing no CTG repeat with (red) or without (blue) *Cre* induction and hygromycin B selection. Data are presented as means ± SD of three independent experiments.
