# Supplementary material for: Expanded CUG Repeat RNA Induces Premature Senescence in Myotonic Dystrophy Model Cells
Source: Front Genet. 2022 Mar 25;13:865811. doi: 10.3389/fgene.2022.865811 (PMC8990169; doi:10.3389/fgene.2022.865811)
Supplement: Supplementary file 2 [file DataSheet1.PDF]

Figure S1

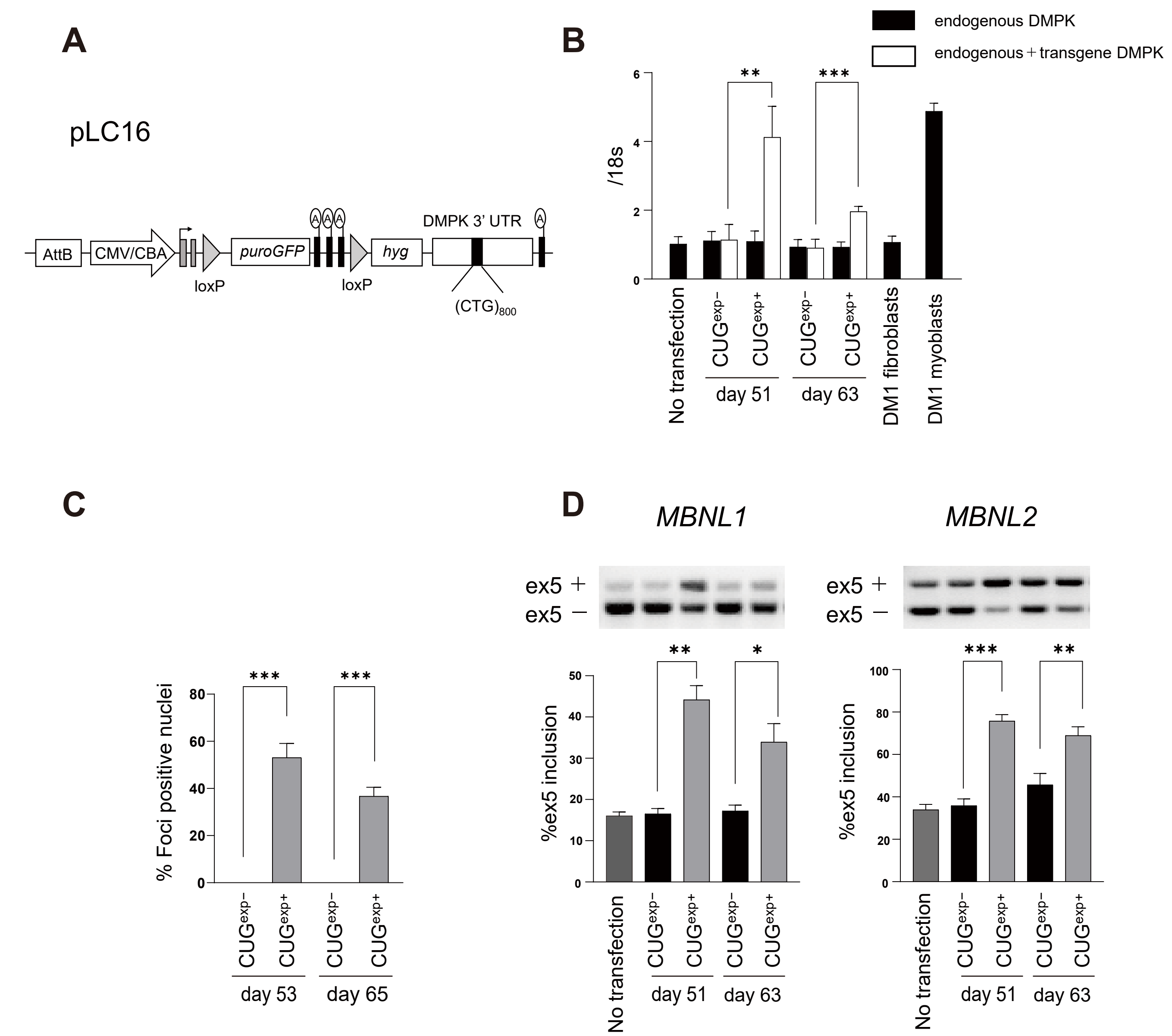

Supplemental Figure S1

(A) Diagram of pLC16, a construct for conditional expression of expanded CTG repeats. Initially this construct expresses the puromycin resistance-GFP (*puroGFP*) selectable marker. The triple 'A' denotes the transcription-terminator element, consisting of three consecutive SV40 polyadenylation signals. Upon *Cre*-mediated excision of the selection cassette and transcription terminator, the construct expresses the hygromycin selectable marker (*hyg*), fused to the 3′-UTR from *DMPK*. **(B)** RNA levels of *DMPK* 3′-UTR (transgene + endogenous) and endogenous *DMPK* in non-transfected TIG-3 cells (no transfection), CUG^{exp-} and CUG^{exp+} cells, DM1 fibroblasts, and DM1 myoblasts, determined by quantitative real-time reverse transcriptase (qRT)-PCR. Mean ± SD. ** $p < 0.01$, and *** $p < 0.001$. **(C)** Histogram showing the percentage of cells with nuclear foci of CUG^{exp} RNA in CUG^{exp-} and CUG^{exp+} cells at days 53 and 65. The number of cells counted was at least 100 for each experiment. Mean ± SD, n = 3 or more. *** $p < 0.001$. **(D)** Analysis of alternative splicing of *MBNL1* exon 5 and *MBNL2* exon 5 by RT-PCR in CUG^{exp-} and CUG^{exp+} cells at days 51 and 63. Top: representative gel images of RT-PCR assays. Bottom: bar graph representation of splicing abnormality. Mean ± SD. * $p < 0.05$, ** $p < 0.01$, and *** $p < 0.001$.
